# Risk Map of Cholera Infection for Vaccine Deployment: The Eastern Kolkata Case

**DOI:** 10.1371/journal.pone.0071173

**Published:** 2013-08-02

**Authors:** Young Ae You, Mohammad Ali, Suman Kanungo, Binod Sah, Byomkesh Manna, Mahesh Puri, G. Balakrish Nair, Sujit Kumar Bhattacharya, Matteo Convertino, Jacqueline L. Deen, Anna Lena Lopez, Thomas F. Wierzba, John Clemens, Dipika Sur

**Affiliations:** 1 International Vaccine Institute, Seoul, Republic of Korea; 2 National Institute of Cholera and Enteric Diseases, Kolkata, India; 3 Indian Council of Medical Research, New Delhi, India; 4 HumNat Lab, Department of Biological Systems Engineering, Virginia Tech, Blacksburg, Virginia, United States of America; 5 Bioinformatics Institute, Virginia Tech, Blacksburg, Virginia, United States of America; 6 Institute for Critical Technology and Applied Science, Virginia Tech, Blacksburg, Virginia, United States of America; 7 Menzies School of Health Research, Casuarina, Northern Territory, Australia; 8 University of the Philippines Manila, National Institutes of Health, Manila, Philippines; 9 University of California Los Angeles, School of Public Health, Los Angeles, United States of America; University of Florida, United States of America

## Abstract

**Background:**

Despite advancement of our knowledge, cholera remains a public health concern. During March-April 2010, a large cholera outbreak afflicted the eastern part of Kolkata, India. The quantification of importance of socio-environmental factors in the risk of cholera, and the calculation of the risk is fundamental for deploying vaccination strategies. Here we investigate socio-environmental characteristics between high and low risk areas as well as the potential impact of vaccination on the spatial occurrence of the disease.

**Methods and Findings:**

The study area comprised three wards of Kolkata Municipal Corporation. A mass cholera vaccination campaign was conducted in mid-2006 as the part of a clinical trial. Cholera cases and data of the trial to identify high risk areas for cholera were analyzed. We used a generalized additive model (GAM) to detect risk areas, and to evaluate the importance of socio-environmental characteristics between high and low risk areas. During the one-year pre-vaccination and two-year post-vaccination periods, 95 and 183 cholera cases were detected in 111,882 and 121,827 study participants, respectively. The GAM model predicts that high risk areas in the west part of the study area where the outbreak largely occurred. High risk areas in both periods were characterized by poor people, use of unsafe water, and proximity to canals used as the main drainage for rain and waste water. Cholera vaccine uptake was significantly lower in the high risk areas compared to low risk areas.

**Conclusion:**

The study shows that even a parsimonious model like GAM predicts high risk areas where cholera outbreaks largely occurred. This is useful for indicating where interventions would be effective in controlling the disease risk. Data showed that vaccination decreased the risk of infection. Overall, the GAM-based risk map is useful for policymakers, especially those from countries where cholera remains to be endemic with periodic outbreaks.

## Introduction

John Snow’s cholera map of 1855 [1] is a disease map on which he plotted the households with cholera deaths in London. A disease map can identify geographic variations of disease incidence, which in turn can be useful to identify areas of unusually high risk requiring preventive action, to formulate etiological hypotheses, and to demonstrate patterns of risk allowing better allocation of resources [2]. As disease variations have a spatial expression, understanding geographical distribution of disease is of considerable importance to public health workers and epidemiologists [3]. Notably, the John Snow’s map revealed that families receiving water from the Broad Street hand pump were more likely to have cholera deaths, which helped the health authorities making appropriate area-based interventions for controlling the disease.

Given advancement of our understanding about transmissions of cholera and its control mechanisms, cholera remains a global health problem [4]. More and more countries are being suffered from the scourge of the disease. One reason could be spatial patterns of risk for cholera in an endemic area are not clearly known, thus an effective control mechanism could have never established in a cholera endemic country. During March and April 2010, there was a cholera outbreak in the slums of eastern Kolkata, India, a cholera endemic area. We investigated the data of a geographically referenced population-based surveillance from a cholera vaccine trial conducted in the area, and plotted the cases during the outbreak in the trial area. We used the surveillance data on cholera and applied a generalized additive model (GAM), which combines smoothing with the ability to analyze binary outcomes and adjust for covariates [5], to identify areas of higher and lower risk for cholera over time. We also evaluated the socio-environmental characteristics between the high and low risk areas of the disease and the impact of the vaccination on spatial risk for cholera.

## Methods

### The Study Area and Data

**Figure 1:**
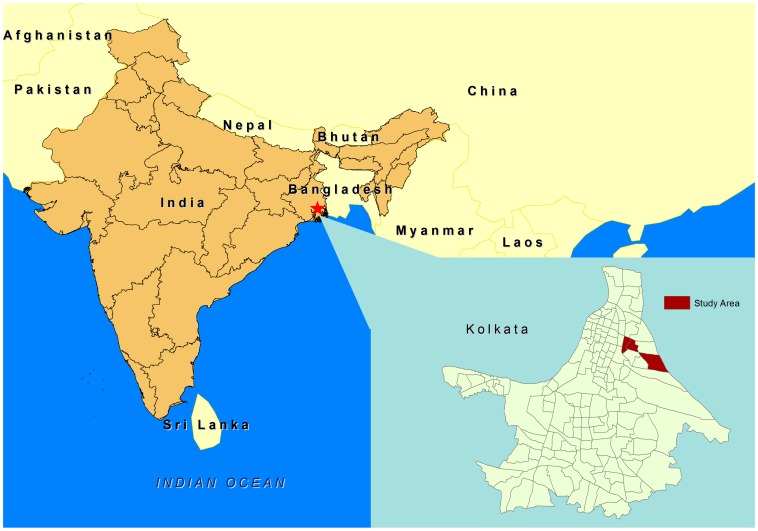
The study area in Kolkata, West Bengal, India.

The study was conducted in urban slum communities in Kolkata, the capital of the state of West Bengal, India (Figure 1). Kolkata is the third largest city in India with 13 million residents into 1,450 km^2^ making it is one of the world’s most densely populated cities. The Municipal Corporation area is demarcated into 141 administrative wards [6]. The study site comprises three contiguous wards: 29, 30 and 33. In the study area, the streets are narrow with little space between houses, piped municipal water supply is intermittent, and several households share one or two latrines and water taps [7]. There is a canal that runs east of ward 29 and south of ward 33. It is one of the main drainages of rain and waste water in the eastern part of Kolkata.

To register the study population, beginning in January 2003, two censuses were conducted in the original study area that includes all of ward 30 and part of ward 29. Subsequently, the study area was expanded in 2005 in preparation for a cholera vaccine trial. Each census captured the *de jure* population defined as persons who stated their residence in the study area was their regular residence and the residence is legally recognized and a registered slum [8] that excluded people who live in high rise building. We also collected socio-demographic information of each household during census survey. The population database was updated monthly though vital demographic events in the study area. Each household in the study area was identified by its geographic coordinates in the geographic information system (GIS) database. The details on how the GIS database was created have been described elsewhere [9].

Nine project health clinics were established in the community to undertake diarrhea surveillance in addition to the two referral hospitals: Infectious Diseases and B.C. Roy Children. Private medical practitioners were encouraged to refer patients with diarrhea to the project health clinics. Patients from the study area were identified by use of household identification cards and a computerized database. Study physicians recorded pertinent clinical details on a structured clinical data form. Rectal swabs were obtained from all patients presenting with history of loose stools and transported in Cary-Blair media to a laboratory at the National Institute of Cholera and Enteric Diseases (NICED) within 8 hours of specimen collection. At the laboratory, rectal swabs were examined for *Vibrio cholerae* by use of conventional method [10].

Vaccination against cholera followed a two-dose schedule administered in two rounds. Each dose of the vaccine contains inactivated *Vibrio cholerae* O1 cells representing the El Tor and classical biotypes and the Inaba and Ogawa serotypes, as well as serogroup O139 cells. The first round was from July 27 to August 13, 2006, and the second from August 27 to September 10, 2006, to ensure a minimum inter-dose interval of 14 days. Residents were eligible to receive a study intervention if they were aged one year or older and were not pregnant. Each agent was given as a two-dose regimen with an inter-dose interval of at least 14 days, and the coverage for the study intervention was about 68% [11]. We classified the study period as pre-vaccination and post-vaccination. The vaccination period (July 27 to September 10, 2006) was excluded. We aggregated one-year data of the pre- and two–year post-vaccination periods by the geographic points of residence in the GIS database for the spatial analysis. Two-year of post-vaccination data were used, because there were only a few cases in the 1^st^ year of post-vaccination period.

### Spatial Models

We used a generalized additive model (GAM) [5] to estimate smoothed log odds as a function of space and converted to odds ratios using whole population as a reference. We modeled location using a bivariate smooth (*S*) of latitude (x) and longitude (y)
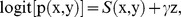
where logit[p(x,y)] is the log of the disease odds at location (x,y), z is a vector covariates, and γ is a vector of parameters. We required the type of smoother and the span size for estimating smoothing term, *S*(x,y). The span size determines over which averaging takes place. Since the population distribution varied in space, we used a locally-weighted regression smoother to adapt to the changes in population density. The amount of smoothing depends on the percentage of the data points in the neighborhood, referred to as the span size. We chose an optimal span by minimizing the Akaike Information Criterion (AIC) from 1% to 99% of the size of study area increased by 1%. Note that small span sizes reduce bias but increase variance, and large span sizes produce smoother surfaces resulting in increased bias and reduced variability.

We created a rectangular grid covering the study area (100 rows and 100 columns) using the minimum and maximum latitude and longitude coordinates of the study area. Grid points lying outside the study area were clipped. We estimated adjusted odds ratios (ORs) at each grid point using whole study area as the reference, dividing the odds at each grid point by the odds calculated by the reduced model omitting the location smoothing term. We permuted the locations of subjects and reran the GAM model 999 times to estimate the distribution of log odds under the null hypothesis at each point. We define areas of significantly decreased odds (“low risk areas”) to include all points that rank in the lower 2.5% of the pointwise permutation distribution and areas of elevated odds (“high risk areas”) to include all points that rank in the upper 2.5% of the pointwise permutation distribution [12]–[14]. We superimpose the 2.5% and 97.5% contour lines on the point estimate map.

In order to make the maps visually comparable, we mapped all results using the blue to red continuous (unclassified) color scale and range of odds ratios, 0.1–2.0. This range covers most but not all of the ORs observed in our analysis. If the ORs is >2.0 we set it to 2.0 and if the ORs is <0.1 we set it to 0.1 for mapping purpose. We used R programming language to run the GAM. Results from the GAM were exported from R into ArcGIS for mapping.

### Statistical Analysis

To evaluate the socio-environmental characteristics of the study population between the high and low risk areas, we used Generalized Estimating Equations (GEE) with the logit link function [15], and adjusted for the household level correlation in the data. The models took living in high or low risk area for each analyzed individual as the dependent variable and fitted several socio-environmental risk factors of cholera detected in earlier studies [11] as the independent variables in models using independent and exchangeable within-household correlation matrices. Coefficients of independent variables in the models were exponentiated to estimate the odds ratio of cholera associated with different levels of the variable. Standard errors for the coefficients were used to estimate p-values and associated 95% confidence intervals (95% CI) for the ORs.

### Ethics and Monitoring

The study protocol was approved by the Drugs Controller General of India, the ethics committee of the National Institute of Cholera and Enteric Diseases, the Health Ministry Screening Committee of India, and the International Vaccine Institute Institutional Review Board. Written informed consent was obtained from residents older than 18 years and from the guardians of residents aged 1 to 17 years of age. Additional written assent was obtained from residents aged 12 to 17 years. The trial was registered at ClinicalTrials.gov number, NCT00289224.

## Results

**Figure 2:**
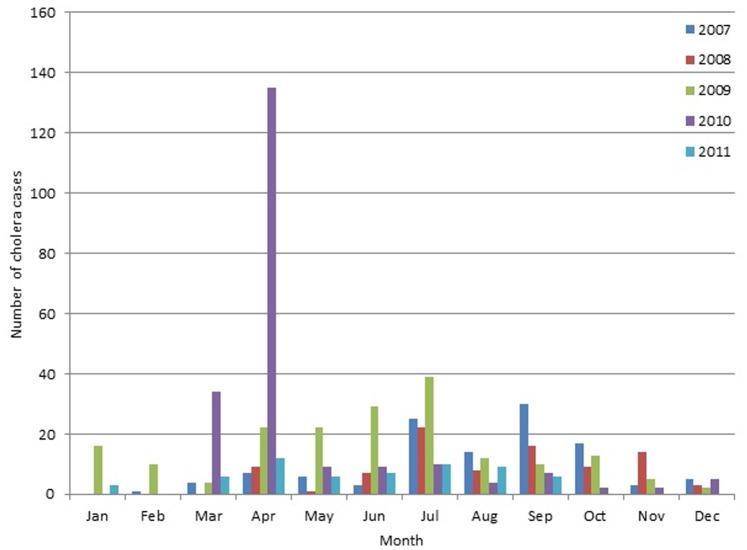
Number of cholera cases by month during 2007–2011 in the study area in Kolkata, India.

**Figure 3:**
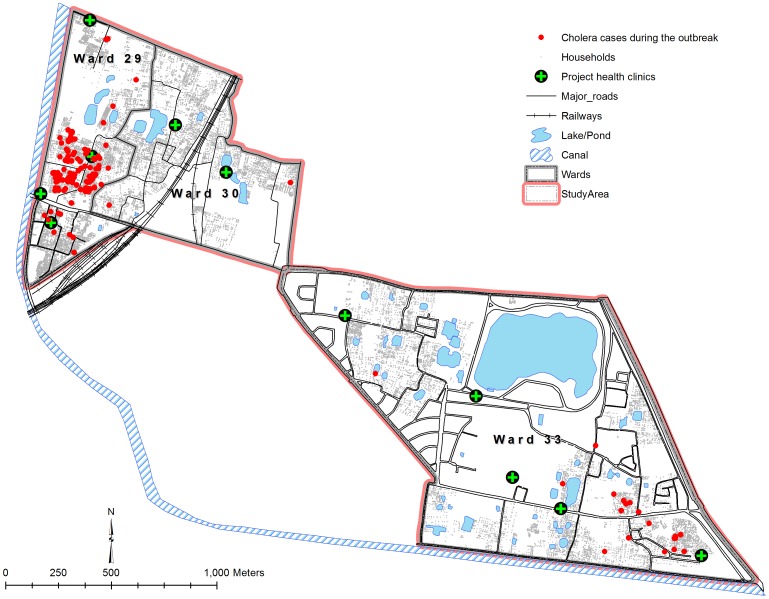
Distribution of the cholera cases in the study area during the large outbreak (March and April 2010) in the study area.

During the one-year pre-vaccination and two-year post-vaccination periods, 95 and 183 cholera cases were detected in 111,882 and 121,827 study participants under surveillance, respectively. There were 169 cases during the large outbreak (March and April 2010) in our study area (Figure 2). The residences of those cases are shown in Figure 3.

**Figure 4:**
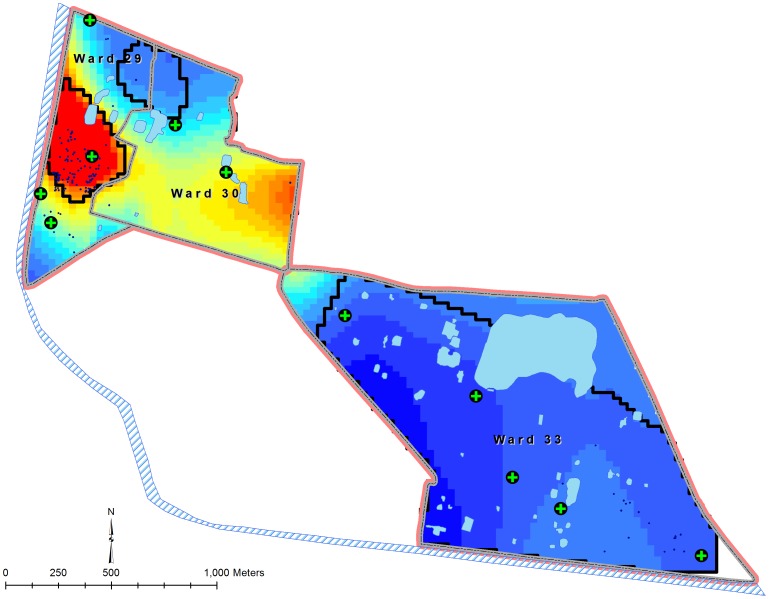
Spatial patterns of risk for cholera during the pre-vaccination period in the study area, Kolkata, India.

**Figure 5:**
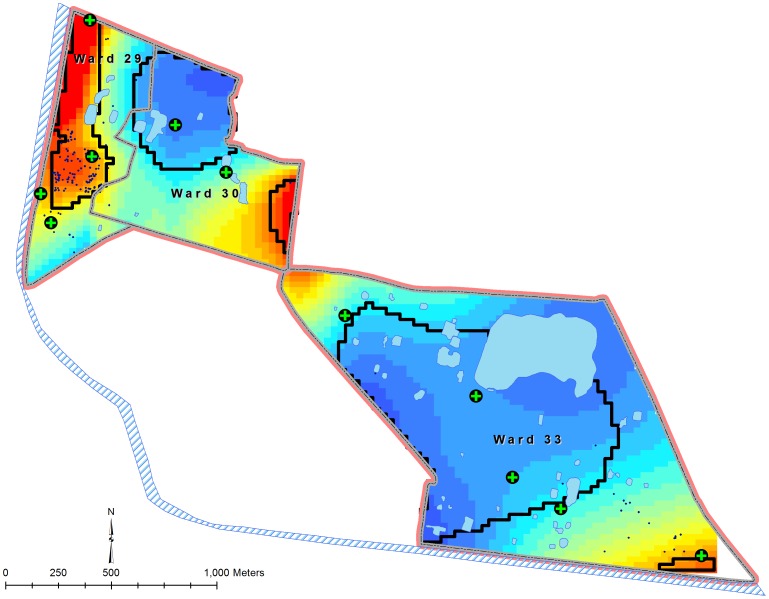
Spatial patterns of risk for cholera during the post-vaccination period in the study area, Kolkata, India.

The results of the GAM model for the pre- and post-vaccination periods are presented in figures 4 and 5. Predicted odds ratios (ORs) during the pre-vaccination period ranged from 0.05 to 4.77, and the global permutation test indicated a statistically significant odds of the disease (p<.001) in the west part of the study where most of the cases during the outbreak were occurred (Figure 4). The predicted ORs during the post-vaccination period ranged between 0.23 and 4.70, and the significantly high risk area for cholera was also observed in the same west part (Figure 5). However, there was a change in the alignment of the high risk area for cholera during the post-vaccination period.

The socio-environmental characteristics of study population between high and low risk areas for cholera are shown in Table 1. In both the pre and post-vaccination periods, the high risk areas for cholera were characterized by people using unsafe water sources for drinking, living in poor socioeconomic status (defined by not living in own house), and living close proximity to the canal. During the post-vaccination period, a significantly lower vaccine uptake was observed in the high risk areas for cholera compared to that in the low risk areas for cholera. The high risk areas for cholera were also characterized by people living in close proximity to the health clinics in both the pre- and post-vaccination periods.

**Table 1:** Socio-demographic characteristics between significantly high and low risk areas in the slum of Kolkata, India.

	Pre-vaccination period			Post-vaccination period		
Variables	High risk (n = 26,354)	Low risk (n = 32,532)	OR* (95% CI; p-value)	High risk (n = 27,191)	Low risk (n = 21,777)	OR* (95% CI; p-value)
Cholera vaccine recipients	**–**	**–**	**–**	11,198 (41.2)	10,798 (49.6)	0.525 (0.475–0.581; <.001)
Using safe toilet	948 (3.6)	6,350 (19.5)	0.904 (0.681–1.201; 0.488)	900 (3.3)	3,540 (16.3)	1.017 (0.799–1.294; 0.893)
Using safe water source (own tap)	1,753 (6.7)	10,311 (31.7)	0.754 (0.596–0.955; 0.019)	1,389 (5.1)	6,391 (29.3)	0.374 (0.311–0.452; <.001)
Using boiled or filtered water	1,084 (4.1)	7,575 (23.3)	0.775 (0.598–1.004; 0.054)	875 (3.2)	5,487 (25.2)	0.271 (0.229–0.321; <.001)
Living in own house	4,496 (17.1)	18,334 (56.4)	0.143 (0.123–0.166; <.001)	4,618 (17)	11,654 (53.5)	0.332 (0.293–0.376; <.001)
Distance (meter) from thehousehold to the canal	189.4 (85.6)	482.4 (361.3)	0.9948 (0.9946–0.9951; <.001)	181.8 (205.5)	678.9 (234)	0.994 (0.993–0.994; <.001)
Distance (meter) from thehousehold to the nearest projecthealth clinic	112.8 (45.4)	247.4 (107.7)	0.979 (0.978–0.980; <.001)	112.8 (59.2)	217.1 (99.3)	0.992 (0.991–0.993; <.001)

Note: Number and per cent of population (in parenthesis) are shown for the dichotomous variables and mean and standard deviation (in parenthesis) are shown for the continuous variables (distances).

* The odds ratio for the cited variable, adjusted for all other variables in the table, in a model using Generalized Estimating Equations (GEE) with the logit link function.

## Discussion

Our results suggest that the outbreak was largely occurred in the areas of high risk for cholera and among the poorest residents of an area located near the canal, which is used as the main drainage for rain and waste water. Usually, an outbreak occurs in an area with immunologically naïve population. The results of our study indicate that the outbreak can even occur in the areas where immune level of the population is believed to be high. Poverty and living adjacent to the canal were consistent characteristics of the high-risk area population. Following the immunization campaign, the geographic alignment of the high-risk area for cholera was slightly changed. We speculate that the geographic realignment may have been brought about by the vaccination campaign that altered population levels of immunity, which in turn may have altered the disease transmission patterns, ultimately changing the spatial patterns of risk for cholera. Similar changes have been reported earlier [16].

We observed that there was significantly lower vaccine uptake in the high risk versus low risk areas for cholera, and, as reported earlier, only one-third of the population received the cholera vaccine [10]. The poor vaccine uptake in the high risk areas suggests high vaccine coverage is required to reduce disease risk. Close proximity to the canal was a consistent attribute of the high risk area. *Vibrio cholerae* were previously found in the canals of Kolkata [17]. It may be the canal water leaks into damaged pipes that carry drinking water [18], which is common in the slum areas [19], and that could have created increased risk for cholera. Close proximity of the high risk area to the health clinic reflects health care behavior of the people, as observed elsewhere [20]–[21]. Therefore, it is important to adjust the bias in a risk analysis.

We used the GAM model to determine high and low risk areas for cholera in the slums of Kolkata, because it is an effective approach for modeling spatial distributions of data, combining a number of desirable features, the ability to estimate odds ratios (ORs) while adjusting for confounders and selection of optimum degree of smoothing [13]. An advantage of the GAM method is that theoretical considerations of bias and variance are used to choose an optimal smoothing span [22]. The GAM model provided us the magnitude of risk at each point in the study area after adjusting for the population density. However, the GAM model is highly computing intensive. It required more than twenty hours running the model in a state-of-the-art computer.

This study shows that the outbreak was largely occurred in the high risk areas for cholera, and the high risk areas are limited to only a small part of the endemic area. These suggest that a control mechanism does not require setting up in all over in that slum area, but can focus only on the high risk areas where it created increased risk of the disease as well as the outbreak. We call this source drying, and if the source drying is effectively done, it is less likely that the disease will be spread in other part of the area. Implementing such source drying control mechanism is feasible to implement in a resource limited country where cholera is endemic. It is worth mentioning the 64^th^ World Health Assembly urges all member states to strengthen cholera control efforts including the use of oral cholera vaccines together with modalities such as water, sanitation, hygiene and early treatment and detection [23]. Considering the limitation of resources in the cholera affected countries, WHO suggest high-risk area based solution in the control mechanism [24]. The findings of our paper and the kind of this cholera map are helpful for the policymakers, especially those from countries where cholera remains to be endemic with periodic outbreaks, for setting up an appropriate intervention strategies within the limited resource of their countries.
