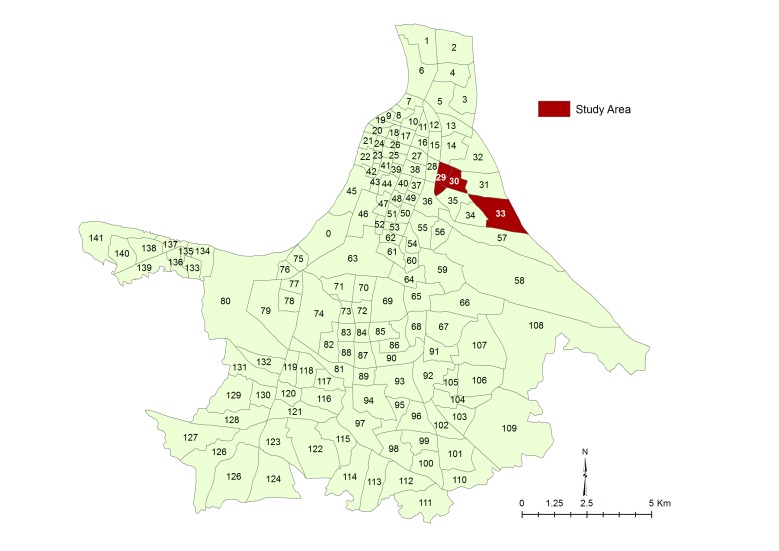# Correction: Risk Map of Cholera Infection for Vaccine Deployment: The Eastern Kolkata Case

**DOI:** 10.1371/annotation/e7cdf42b-e85a-4b94-bf1c-66e35ca136ca

**Published:** 2013-10-11

**Authors:** Young Ae You, Mohammad Ali, Suman Kanungo, Binod Sah, Byomkesh Manna, Mahesh Puri, G. Balakrish Nair, Sujit Kumar Bhattacharya, Matteo Convertino, Jacqueline L. Deen, Anna Lena Lopez, Thomas F. Wierzba, John Clemens, Dipika Sur

The authors have noted that the map of India in Figure 1 is not authentic. They therefore remove the map from the figure. The correct Figure 1 can be viewed here: 

**Figure:**