# Correction: Risk Map of Cholera Infection for Vaccine Deployment: The Eastern Kolkata Case

**DOI:** 10.1371/annotation/537e3894-e768-44bf-a1ae-3a185b29a81f

**Published:** 2013-11-15

**Authors:** Young Ae You, Mohammad Ali, Suman Kanungo, Binod Sah, Byomkesh Manna, Mahesh Puri, G. Balakrish Nair, Sujit Kumar Bhattacharya, Matteo Convertino, Jacqueline L. Deen, Anna Lena Lopez, Thomas F. Wierzba, John Clemens, Dipika Sur

It has come to the attention of the PLOS ONE editors that Dr Matteo Convertino, who handled the manuscript as Academic Editor, is also one of the listed authors for this article. In line with the PLOS ONE competing interests policy (http://www.plosone.org/static/competing.action), we consider this as a potential competing interest and thus the declaration of Competing interests provided with the published article is inaccurate.

The version of the manuscript originally submitted to PLOS ONE did not include Dr Convertino as an author. Dr Convertino handled the peer review process for the submission and after an initial editorial decision had been reached, the authors approached Dr Convertino regarding the revisions to be undertaken on the manuscript and requested his assistance in relation to the analysis of the data and the presentation of the work. Dr Convertino actively collaborated with the authors in the revisions and contributed to the design of the revised modeling analyses, figure editing, analysis and plotting of the results and writing of the revised manuscript. In the light of these contributions, the authors invited Dr Convertino to become an author on the revised manuscript.

While the editors consider that Dr Convertino's contribution fulfills the journal's criteria for authorship, his involvement in the preparation and editorial handling of the revised manuscript constitutes a potential competing interest, and thus, the revised manuscript should have been handled by a different Academic Editor.

The authors have apologized for breaching journal policy in relation to competing interest disclosure and for not disclosing to journal staff that they had invited the Academic Editor to join the authorship byline on this study.

In the light of this potential competing interest, the PLOS ONE editors have asked an independent member of the editorial board, Martyn Kirk, to carefully evaluate the peer review process of this article. Dr Kirk has confirmed that the final decision to publish was acceptable but considered that some additional information should be supplied in relation to specific methodological aspects of the study. This information does not alter the results and conclusions of the article. The authors have now provided the requested methodological detail which they are also reporting as part of this Correction:

A generalized additive model was used to estimate smoothed log odds as a function of space and converted to odds ratios using whole population as a reference. In the model, latitude and longitude as a smoothed, interaction term (s(x,y)) were used to account for spatial autocorrelation in the data. A locally-weighted regression smoother was used and the span size of the smoother was determined by minimizing the Akaike Information Criterion from 1% to 99% of the size of study area increased by 1%.

The study was derived from our assumption that outbreaks occur in the high-risk area for the disease. The authors used one-year data of the pre-vaccination and two- year data of the post vaccination period to define the high-risk areas. The vaccination period was July 27 to September 10, 2006. The data from the outbreak period (March and April 2010) were not included to create the high-risk areas, these data were used to evaluate whether the outbreak cases fall within the high-risk areas or not. Vaccine coverage was found to be significantly lower in the high-risk areas compared to low-risk areas. 

